# Remarks upon the Organization of the Medical Department of the Army and the Effects of Marching and a Camp Life in Producing and Modifying Disease

**Published:** 1847-08

**Authors:** W. B. Herrick

**Affiliations:** Professor of Anatomy in Rush Medical College, late Surgeon 1st Regiment Illinois Volunteers, &c.


ARTICLE VII.

*Remarks upon the Organization of the Medical Department of the Army, and the Effects of Marching and a Camp Life in producing and modifying Disease.* By W. B. Herrick, M.D., Professor of Anatomy in Rush Medical College, late Surgeon 1st Regiment Illinois Volunteers, &c.

In compliance with the solicitations of many friends, and with the view of satisfying, to some extent, the desire manifested by our medical brethren for information concerning the medical department of our army in Mexico, we propose to lay before our readers, from time to time such facts and remarks as we may deem most interesting and worthy of their attention.

As an introduction to our subject, we shall endeavor, at this time, to give a brief history of a surgeon's duties, as prescribed by army regulations, and to make a few remarks of a general character upon the effect of long marches and a camp life in producing and modifying disease.

With regard to the organization of the medical department, it may be stated that the Surgeon General, stationed at Washington, is charged with its administrative details, and has the control of all the officers belonging to it. He assigns the surgeons and assistant surgeons to regiments, posts, and stations, and all official communications from them are made direct to him.

The senior medical officer of every separate division of the army, in the capacity of medical director, inspects hospitals, sees that the necessary medicines are provided, and that the surgeons and assistant surgeons perform their duties and abide by the rules and regulations given for their government and direction.

The surgeon of a regiment, so far as his professional duties are concerned, obeys the instructions of the medical director, and is responsible to him for the order and neatness of his hospital, for the manner in which the assistants and attendants perform their duties, and for the comfort and proper treatment of the sick. It is his duty to keep, for the inspection of the medical director, a register containing the names and rank of the sick under his charge, and also, a prescription and diet book containing his written prescriptions and directions for every case.

It is the duty of the orderly sergeant of each company to make, to the surgeon, written morning reports of sick, and to see that those who are able, present themselves for inspection at time and place appointed by the surgeon. Every surgeon or assistant surgeon, having charge of sick, makes monthly reports to the medical director, and every one attending to the sick of a regiment, post, or garrison, makes daily morning reports to the commanding officer with such remarks and suggestions in relation to whatever may effect the health of the soldiers as he may deem necessary and proper.

With regard to personal observation and experience, it may be stated that the writer of this communication joined the division of the army under General Wool, at San Antonio, Texas, about the first of September last, and was assigned to duty with the 1st Regiment Illinois volunteers, under the lamented Colonel Hardin, about the 20th of the same month, and continued with that regiment, for the most of the time, the only medical officer until the division joined the main army under General Taylor, near Saltillo, about the first of January of the present year.

It appears from our monthly reports, that from the 20th of September to the 1st of October, the whole number of cases treated in the regiment—the mean strength of which was, at that time, about 800—was as follows:

| | |
|---|---|
| Remaining sick on the 20th, - - - - | 30 |
| Taken sick after the 20th and before the 1st of Oct.— | |
| Of Miasmatic Fever, - - - - - - | 45 |
| Diarrhœa, - - - - - - | 38 |
| Dysentery, - - - - - - - | 2 |
| Bronchitis, - - - - - - | 20 |
| Pneumonia, - - - - - - - | 3 |
| Ulcers, - - - - - - - | 3 |
| Abscess, - - - - - - - | 3 |
| Cutaneous Eruption, - - - - | 4 |
| Fracture, - - - - - - - | 1 |
| Total, - - - - - - - | 119 |
| Aggregate, - - - - - - - - | 149 |

It will appear, by comparing the above report for a part of September with those of other months which will follow, that the number of sick was much greater in proportion than at any period of time after, of the same length.

Among the causes which conspired to produce a greater amount of sickness at this time than at any subsequent period, the following may be mentioned as, probably, among the most prolific:

The men had then been in service but a short time, and were suffering temporarily, from a change of diet and habits consequent upon exchanging the pursuits of civil life, and the comforts of home, for the duties of soldiers and the exposures incidental to a camp life. It is well known, also, that about a month previous to this time, the 1st and 2d Illinois Regiments, soon after the measles had prevailed as an epidemic in their camp, made the march from the coast to San Antonia just at the close of the rainy season, under a scorching sun, and over plains covered, in many places, to the depth of two or three feet with water. The effect of this first long march was most disastrous. Many convalescent from measles had relapses; others contracted severe bronchial affections or diarrhœa, and all suffered more or less from exposure and fatigue.

The above is the only instance in which marching proved detrimental to health. At every subsequent period during the campaign, the number upon our sick report diminished most rapidly, during and immediately after a long march, and as certainly increased after remaining in camp for any length of time.

Upon referring again to our sick report for the month of September, it appears that the 149, which was the aggregate, as before stated, were disposed of as follows:

| | |
|---|---|
| Returned to duty, - - - - - - - | 95 |
| Left in General Hospital at San Antonio, - - | 40 |
| Discharged from Service, - - - - - - | 5 |
| Died, - - - - - - - - - | 1 |
| Remaining on the sick list but convalescent and able to march, - - - - - - | 8 |

It is a remarkable fact, that those of the sick who, in compliance with their own requests, were permitted to remain with the regiment and participate in the fatigues and exposures incidental to this long march, recovered more rapidly and were sooner able to return to duty than others apparently as well but who were left behind in hospital.

It appears that of the one hundred and forty-nine treated during the last ten days of September, ninety-five were returned to duty before the first of October, and previous to marching. Most of these were convalescents from the different diseases above mentioned, who had been recovering but slowly whilst they remained still and quiet in camp, yet after a few days march, they improved most rapidly, and after a week or two were able to perform a day's march with as much ease apparently, as others. Protracted diarrhœa and bronchial affections, which had yielded but slowly under the most approved modes of treatment in camp and hospital, were apparently cured without medicine, by active exercise in the open air.

These facts, as it appears to us, show that the advantages to be derived from this kind of treatment have been, in general, underrated by practitioners. From observing during the past year, the salutary effects of long marches in preventing and curing disease, we have become convinced that moderate and even active exercise in the open air, without medicine, is more beneficial in many diseases than the most approved therapeutic remedies administered to patients inactive and confined in badly ventilated apartments. By comparing the following report for the month of October, during which we were, for the most of the time, on the march from San Antonio to Monclova, with that previously given of the last ten days of September spent in camp, we find the difference in the number of cures most remarkable.

As before stated, eight remained sick the 30th of September. During the month of October the number added was as follows:

| | | |
|---|---|---|
| Cases of | Intermittent Fever, | 48 |
| | Remittent Fever, | 41 |
| | Diarrhœa, | 42 |
| | Mumps, | 4 |
| | Bronchitis, | 4 |
| | Sprains, | 4 |
| | Neuralgia, | 1 |
| | Inflamed Testis, | 1 |
| | Ophthalmia, | 1 |
| | Scorbutis, | 1 |
| Total, | | 147 |

Aggregate one hundred and fifty-five. These cases were disposed of during the month as follows:

| | |
|---|---|
| Returned to duty, | 119 |
| Sent to Hospital, | 10 |
| Remaining sick, | 10 |
| Convalescent, | 16 |

Thus it appears that the whole number taken sick during all of October was but one hundred and forty-seven, making forty-nine the proportion for ten days, whilst on the march; and one hundred and nineteen for the same length of time in September whilst in camp.

The importance of keeping an army constantly moving in order to secure the health of soldiers is made still more apparent by referring to our report for January made soon after we had joined general Taylor's advance near Saltillo, after having performed the almost unprecedentedly rapid and long march of about one thousand miles:

Taken sick during the month—

| | |
|---|---|
| Of Intermittent Fever, | 11 |
| Remittent Fever, | 23 |
| Diarrhœa, | 9 |
| Constipation, | 10 |
| Hepatic Disease, | 1 |
| Bronchitis, | 4 |
| Pneumonia, | 1 |
| Laryngitis, | 1 |
| Syphilis, | 1 |
| Abscess, | 4 |
| Ulcers, | 3 |
| Fracture, | 1 |
| Rheumatism, | 1 |
| Tumors, | 6 |
| Scorbutis, | 3 |
| Fistulo in Ano, | 1 |
| Anasarca, | 1 |
| Total, | 81 |

From the above statement it appears that the number taken sick during the month of January, as compared with September was as 1 to 4, nearly; and as compared with October as 1 to 1·7.

That the health preserving influence of a long march is very great is made very apparent by this comparison of some of our monthly reports. Still, however, the statement, as made above, falls far short of exhibiting the full extent of its beneficial effects; for during a part of the months both of October and January, we were in camp, and consequently not experiencing, during the whole time, the beneficial effects of the march.

The comparison of some of our daily morning reports, exhibiting the number of cases treated from day to day whilst in camp, with others made during some of our long marches being free from the above objection, and therefore more strictly in accordance with facts, exhibits a still more remarkable difference.

From the first to the tenth of November inclusive, during which time we remained in camp, at Monclova, our daily morning report ranged from nineteen to thirty-two, averaging about twenty-five for each day. From the eighteenth to the twenty-fifth of December, on the other hand, whilst performing the rapid march from Panas to Saltillo, our morning reports ranged from six to eleven, averaging about nine per day.

The comparison of our monthly reports, for reasons before stated, makes the difference appear less than it really was. On the contrary, our morning reports whilst in camp compared with those made upon the march, make it greater than the facts will justify, as it was our practice, upon starting on a long march, to leave our worst cases behind in hospitals. In accordance with this practice, eleven of the worst cases in our regiment were left at Monclova when we left there for Panas, and nine at Panas when we left there for Saltillo.

After making all these allowances, and even admitting that the whole number left behind in Hospital would have remained upon the sick report during the march, had they continued on with the regiment, and still it will be seen that the amount of sickness whilst upon the march would have been nearly one hundred per cent. less than when in camp.

The preceding facts, as it appears to us, show that the difference in the amount of sickness occurring in camp and upon the march, is so great as to make it a matter of vital importance to our army, as well as a subject of interest to the profession.

As to the causes of this great difference, we may remark that the simple diet used upon the march, being less stimulating and irritating than the mixed food more readily obtained in camp, had a less tendency to produce diarrhœa and dysentery. The air, too, in and around large encampments, becomes, after a time, vitiated, and tends really, no doubt, to the production of miasmatic and other diseases. But, above all, inactivity, both of body and mind, produces lassitude and general debility.

Our remarks upon the above subject have already extended too far, perhaps, to be profitable to our readers; we shall therefore close for the present, remarking in conclusion that it is our intention to lay before our readers in the next number of our Journal, a brief history of some of the most important cases that have occurred under our observation; and also a short account of our surgical experience during and after the battle of Buena Vista.

